# Fordyce Happiness Program and Performance for Mothers of Children with Cleft Lip and Palate Referring Healthcare Team in Isfahan University of Medical Sciences in 2015

**Published:** 2017

**Authors:** Zeinab HEMATI, Samira ABBASI, Parastoo OUJIAN, Davood KIANI

**Affiliations:** 1 Nursing and Midwifery Care Research Center, Isfahan University of Medical Sciences, Isfahan, Iran.; 2 Department of Psychiatric Nursing, Faculty of Nursing and Midwifery, Isfahan University of Medical Sciences, Isfahan, Iran**.**; 3 Nursing and Midwifery Care Research Center, Isfahan University of Medical Sciences, Isfahan, Iran.; 4 Hajar hospital, Psychiatric ward, Shahrekord University of Medical Sciences, Shahrekord, Iran**.**

**Keywords:** Fordyce happiness program, Performance, Mothers, Children, Cleft lip and palate

## Abstract

**Objective:**

The present study was conducted to investigate the effect of happiness program on performance for mothers of children with cleft lip and palate.

**Materials & Methods:**

In this semi experimental study, 64 mothers of children with cleft lip and palate referring healthcare team in Isfahan University of Medical Sciences, Iran were enrolled by simple random sampling in 2015. They were divided to two groups of control and intervention. Then, the program of happiness training was implemented within 10 sessions of two hours each and performance questionnaire were filled out prior two months after the last session. The data were analyzed by paired *t*-test, independent *t*-test, chi-square and Mann-Whitney.

**Results:**

Mean age of the mothers in intervention and control groups was 33.3±6.3 and 33.5±5.8 yr, respectively. Mean age of the children in intervention and control groups was 6.34±3.37 and .03±3.36 yr, in that order. No significant difference was seen on demographic variables between the two groups. Besides, no significant difference was noticed on mean score of performance domains in the two groups before training. However, after 2 months a significant difference on mean score of performance domains was observed (*P*<0.000). Intervention group showed significant differences on mean score of performance domains before and after intervention (*P*<0.000). However, the control group had no significant change.

**Conclusion:**

Given the effect of happiness, program in promoting mothers performance for children with cleft lip and palate, this program can be used in healthcare centers to empower mothers and enhance performance in taking care of children.

## Introduction

The announcement, prenatally or at birth and development of mother-infant interaction, psychosocial development, feeling of attachment between mother-infant, and child's dependency on mother begin. Meanwhile, certain factors such as infant's physical attraction that can greatly affect its development and caretaking are highly important (1). Moreover, oral cleft is the most common superficial deformities of jaw and mouth (2). In the United States, the prevalence of cleft lip is 1/1000 live births, and the prevalence of cleft palate is 1/2000 live births (3). In Iran, various incidence rates were reported (3.77-77/1000 live births) for this anomaly in different regions (except west Iran) (4).

A child's suffering from this anomaly is associated with certain problems such as feelings of shocked, sadness, and guilt that can disturb family's balance (5). The presence of a child in need of special care in a family affects other family members' mental health (6) and lifestyle (7). 

These conflicts and stresses challenge family's health and increase risk for dysfunctional in family performance (8). Family performance is one of the important indicators for quality of life and mental health (7). It refers to family's ability to coordinate and cope with the changes caused throughout lifetime, resolve conflicts, develop attachment among family members and a successful disciplinary pattern, observe interpersonal boundaries, implement principles and regulations of family to protect the whole family system (8). 

Child's acquisition of disease is associated with reduced adaptability and family performance. The adaptation was lower in parents of children with cleft lip than in those of healthy children (9). Moreover, a high proportion of parents with liver disease were dysfunctional family (10). Over half of families with children suffering from cancer were dysfunctional and experienced severe conflicts in caring for their children (11). Since the mothers of these children are more seriously involved in the process of the growth, development, and care to take of their children, they experience higher levels of stress and family conflicts, which can decline their self-efficacy (12, 13).

Therefore, adapting to ongoing conditions is considered the only approach to cope with family conflicts and declined performance (14). Training methods of living happy life is an effective approach to increase adaptation among mothers. Happiness refers to frequently experiencing pleasant emotions, relative absence of unpleasant emotions, and a general feeling of life satisfaction. Happy people have been found better healthy habits, fewer days of illness, tend effectively to communicate with others, be more appreciative and creative, and have healthy thinking styles (15). 

“Relevantly, positive group psychotherapy was effective in promoting mental health and led to enhanced happiness among mothers of children with special needs” (16). Fordyce findings on his model developed to increase happiness were representative of happiness development and mental health promotion. Fordyce happiness program results in certain changes in cognitive and emotional conditions and helps people adopt a more positive attitude toward recent life events and respond to conditions and situations in a positivist and adaptive manner (17). 

Based on the all problems of mothers with mental health and adaptation to their children's disability, which bring about significant outcomes such as declined marital and parent-child relationships and psychosocial health as well as family dysfunction, this study investigated the effect of Fordyce happiness program on performance of the mothers of children with cleft lip and palate.

## Materials and Methods

This semi experimental study was conducted from Mar 2015 to Dec 2015. Aample size was calculated as 32 in each group based on this formula: (d=. /7s^2^, α=0.05, β=0.2) (18).

Research and Technology Deputy of Isfahan University of Medical Sciences (No. 293076) issued ethical approval. The researcher completed pretest questionnaires after described purposes for participants and received written consent from them.

The study was conducted at Cleft Lip and Palate Clinic of Faculty of Rehabilitation, Isfahan University of Medical Sciences, Isfahan, Iran. The patients were enrolled by simple random sampling, the first referring individual with inclusion criteria was assigned to intervention group and the second to control, continuing until the desired number of participants were included in the two groups. Because dropout was likely, first, 40 people were enrolled in the intervention group. Then, because of not attending more than two sessions, some of them were excluded. 

Inclusion criteria were Iranian nationality, literacy, ability to discuss and debate in training classes, and children with cleft lip and palate with age range of 0-12 yr (because of psychological effects on family due to an affected child within this age range (19), and no previous participation in similar training. The exclusion criteria were not attending two consecutive and/or one fourth of all sessions, withdrawing from participation in the study, and complete mental health and consciousness (emergency severe conditions throughout the study).

The training sessions were scheduled and the classrooms of Faculty of Rehabilitation were appointed as location to hold sessions with participants’ consent in the intervention group.

Consisting of eight cognitive components and six behavioral components, teaching material was offered according to Fordyce approach. The material for each session was as follows (20):

1) Participants’ getting acquainted with each other, reviewing sessions’ structure, relevant regulations, protocol, and training techniques of getting more active; 2) Techniques of enhancing social relationships and intimacy; 3) Techniques of expressing emotions and developing optimism and positive thinking, 4) Techniques of decreasing expectations and appreciating, 5) Living at present, 6) Techniques of giving value to happiness and resolving problems and negative emotions, 7) Techniques of discontinuing worries, 8) Techniques of enhancing creativity, 9) Techniques of planning and organizing daily activities; and 10) Filling out the questionnaires two months after the ninth session. The training was done by a psychologist as group and individually through speech, brainstorming and educational aids such as Power Point within 2-h session a week. In addition, the participants were given researcher’s phone number of demanding further advice and support during the two-month follow-up if necessary. Therefore, the patients could call if they had any questions. After the following-up, the patients in both groups filled out the questionnaire again. 

The researcher trained the patients in both groups similarly after filling out the questionnaires. The time needed to fill out the questionnaires was determined two hours for both groups before and after the intervention.

The data were gathered by a demographic data questionnaire and the Family Performance Questionnaire. This scale, derived from McMaster's Model, was developed to describe the structural and organizational features of the family. It addresses six specific domains and one general domain of family performance consisting of problem-solving, relationship, roles, emotional support, emotional conflict, behavioral control, and general performance. This questionnaire consists of 53 four-point scales (from absolutely agree to absolutely disagree) items. Higher scores in any domains represent better performance.

The validity of this questionnaire has already been confirmed by concurrent validity in the studies conducted in Iran. The Cronbach’s alpha coefficient of this scale has already been reported to be 0.77-0.93 for Iranian population (21, 22). The data were analyzed by descriptive and analytical statistics (paired *t*-test, independent *t*-test, Pearson correlation coefficient) in SPSS ver. 20 (Chicago, IL, USA). The level of significance was considered <0.05.

## Results

Mean age of the mothers in treatment and control groups was 33.3±6.3 and 33.5±5.8 yr, respectively and for children was 6.34±3.37 and 5.03±3.36 yr. Chi-square and U-Mann Whitney test showed no significant differences in demographic variables between two groups [*P*≥0.05].

Overall, 62.5% and 56.3% of the children were boys in the intervention and control groups, respectively. The education level of 50% and 46.9% of the women in the intervention and control groups was high school completion certificate, respectively. Besides, 93.7% and 87.5% of the women in the intervention and control groups were homemakers, respectively (Table 1).

Independent *t*-test showed that there was no significant difference for the mean score of performance domains between the two groups before the intervention. However, after the intervention, there was significant difference on the mean score of performance domains between the two groups. The intervention group's mean score of performance domains was better than the control group's after the training (*P*<0.05).

Paired *t*-test showed a significant difference on the mean score of performance domains before and after the training (*P*<0.05). However, this score did not change significantly in the control group (Table 2, 3).

Pearson's and Spearman's correlation coefficients indicated that in the intervention group, the mean performance score of the mothers was significantly associated with children's gender and mother's occupation (*P*<0.05), and not significantly associated with children's age, mothers' age and education, and number of children. In addition, in the control group, the mean performance score of the mothers was significantly associated with children's age, number of children and mother's age, and not significantly associated with children's gender and mothers' education and occupation (*P*>0.05) (Table 4).

**Table 1:** Demographic characteristics in the two groups

**Demographic variables**	**Intervention group**	**Control group**	***P***
Mother Mean age (yrs.) ±SD	33.3±6.3	33.5±5.8	0.8
Child Mean age (yrs.) ±SD	6.34±3.37	5.03±3.3	0.12
Child Sex N (%)			
Male Female	20(62.5) 12(37.5)	18(56.3) 14(43.7)	0.7
Mother Education level N (%)			
Elementary Secondary Diploma University degree	3(9.4) 4(12.5) 16(50) 9(28.1)	6(18.8) 6(18.8) 15(46.9) 5(15.5)	0.3
Mother Employment status N (%)			
Housekeeper Employment	30(93.7) 2(6.3)	28(87.5) 4(12.5)	0.6
Number of children N (%)	2.03±0.73	2.0±0.84	0.8

**Table 2:** Comparison mean score of performance domains in the two groups after training

** Group**	**Intervention**	**Control**	**Independent ** ***t*** **-test results**
**Domains**	**(m**±**sd**)	**(m**±**sd**)	
Emotional support	18.21 ±3.11	14.99 ±3.90	*P*=0.001
Communication	21.21 ±2.12	18.28 ±3.81	*P*<0.000
Problem solving	15.28 ±2.66	12.33 ±3.04	*P*<0.000
Emotional conflict	24.70 ±4.50	21.04 ±4.15	*P*=0.001
Behaviour control	23.78 ±3.78	21.55 ±4.42	*P*=0.03
Role	24.66 ±3.75	22.76 ±3.32	*P*=0.03
General performance	127.8 ±12.36	110.9 ±14.35	*P*<0.000

**Table 3:** Comparison means score of performance domains in intervention group, before and after training

** Time**	**Before**	**After**	**Paired** *** t*** **- ** **test results**
**Domains**	**(m**±**sd**)	**(m**±**sd**)	
Emotional support	16.33 ±3.50	18.21 ±3.11	*P*=0.03
Communication	19.50 ±3.14	21.21 ±2.12	*P*=0.01
Problem solving	13.03 ±2.23	15.28 ±2.66	*P*=0.001
Emotional conflict	21.31 ±5.06	24.70 ±4.50	*P*=0.007
Behaviour control	23.09 ±3.59	23.78 ±3.78	*P*=0.002
Role	22.85 ±3.50	24.66 ±3.75	*P*=0.04
General performance	116.1±11.25	127.8 ±12.36	*P*<0.000

**Table 4:** The association between demographic characteristics and mean performance score in two groups

** Group**	**Intervention**		**Control**	
**Variables**	Statistical result		Statistical result	
Child age	*P*=0.05	r= 0.75	*P*=0.004	r= 0.98
Child gender	*P*=0.02	r=0.88	*P*=0.31	r= 0.83
Number of children	*P*=0.27	r=0.13	*P*=0.01	r= 0.95
Mother age	*P*=0.34	r=0.05	*P*=0.05	r= 0.77
Mother education	*P*=0.15	r=0.39	*P*=0.44	r= 0.01
Mother Occu*P*ation	*P*=0.04	r=0.81	*P*=0.14	r= 0.43

## Discussion

This study was conducted to investigate the effect of Fordyce happiness program on the performance of the mothers of children with cleft lip and palate. An important finding in this study was significant difference in the scores for different domains of family performance between the two groups such as communication, problem-solving, role, emotional support, emotional conflict, behavioral control, and general performance increased significantly in the intervention group compared to the control group. Consistently, happiness program had significant effect in reducing the difficulties associated with emotional regulation such as promotion of physical health and improvement of happiness, appropriate occupational and educational function, social interactions, and interpersonal performance (17). 

Besides, happiness training were performed according to certain approaches such as increasing positive thinking and optimism, decreasing levels of expectations and dreams, and taking a positivist approach to deal with events, greatly affected power of planning for and organizing affairs, and function generally among women (23). 

Families of children with chronic disease were suffering from disturbed family performance (24). Moreover, there are weak performances of parents at all domains in families of children with kidney cancer (7). In the present study, the mothers, governing a tunnel of problems associated with parenting a child with cleft lip and palate, were engaged in a process that declined their performance. Parenting a child with cleft lip and palate could affect different domains of psychosocial and economic status of the parents (25).

Happiness program resulted in improved performance of the mothers at different domains through changing their attitudes toward the world and own abilities, encouraging them to seek out children's abilities and positive capabilities, pay attention to positive aspects of life. Accordingly, the women were encouraged to recognize themselves and their own positive experiences better and figure out the positive role of these experiences in increasing self-respect at domains of communication and role and training positive thinking techniques and skills such as reinforcing and improving positive communication with self and others (26). 

Moreover, happiness training increases the likelihood of positivist perceptions from self and others through promoting the attention paid to positive facts and experiences in the past and helps people recognize their own and others' abilities, capabilities, and positive aspects (27).

The mean performance of the mothers increased significantly in the intervention group compared to the control group. Governing emotional conditions such as shock, feeling guilty, and mourning in these mothers make them sensitive to others' reactions (28) such that through developing negative emotions and mentally setting aside positive ones, they become more ashamed and restless and ultimately trapped in a vicious circle that cannot easily exit from (29).

However, attending happiness-training sessions, encouraging women to seek out their children's abilities and capabilities and pay attention to positive aspects of life. In this study, training how to stop concerns and the techniques of resolving problems and eliminating negative emotions led to improved performance at emotional support and emotional conflict among the mothers. Besides, the mean score for general performance was significantly associated with children's age, gender, and mothers' occupation in the intervention group. 

Family performance was associated with children's age, children's gender, number of children, and parents' education level and economic status (7) such that family performance was affected as the child grew older and experienced new steps of life, potentially causing different situational and evolutionary crises for the family (30). 

The most remarkable limitation of the present study can be failure to schedule the training sessions according to the mothers' working hours. Since lack of attending more than two sessions was considered one of the exclusion criteria, some participants were very likely to be excluded. Therefore, the researcher held the sessions on the weekends to let most participants attend the sessions. Small sample size was another limitation of this study; therefore, future studies must be conducted with a higher sample size. 

**In Conclusion, **in the light of the effect of Fordyce happiness program in enhancing performance of the mothers of children with cleft lip and palate, this intervention can be used for the mothers of children with other special needs, as well. Besides, future studies are recommended to enroll fathers.
